# First reported *Rhodotorula mucilaginosa* brain abscess: Found as coinfection in woman with common variable immune deficiency

**DOI:** 10.1002/ccr3.7896

**Published:** 2023-10-17

**Authors:** Arjun Bhatt, Melinda S. Dunlap, Han Pham, Kimmo J. Hatanpaa, Philip Boyer, Rita M. Gander, Anna G. Symmes

**Affiliations:** ^1^ Department of Internal Medicine Brody School of Medicine Greenville North Carolina USA; ^2^ Departments of Internal Medicine University of Texas Southwestern Medical Center Dallas Texas USA; ^3^ Pathology University of Texas Southwestern Medical Center Dallas Texas USA

**Keywords:** brain abscess, CVID, *nocardia*, *rhodotorula*

## Abstract

*Rhodotorula* is a rare pathogen seen in the immunocompromised host; while cases of *Rhodotorula* meningitis have been reported, there are no published cases of *Rhodotorula* brain abscess. We describe the diagnosis and management of a woman with common variable immune deficiency presenting with concomitant *Rhodotorula* and *Nocardia* brain abscesses.

## Graphical abstract

In a patient with newly diagnosed common variable immunodeficiency and clinical symptoms, brain MRI demonstrated intra‐axial ring‐enhancing lesions with restricted diffusion and surrounding edema. These lesions were revealed to be Nocardia and Rhodotorula brain abscesses.

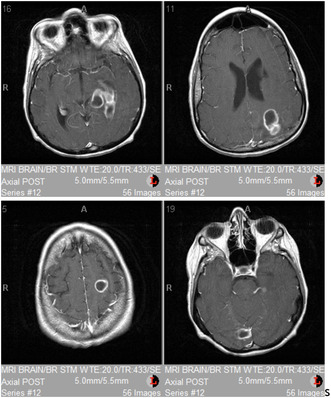

## 1 INTRODUCTION

*Rhodotorula*, a pink to red‐pigmented yeast in the family *Sporidiobolaceae*, is considered a ubiquitous commensal organism. Previously, *Rhodotorula* was not considered a human pathogen, but with the increasing prevalence of infectious niduses (e.g., central venous catheters), *Rhodotorula* has since emerged as a cause of opportunistic infection with a prevalence between 0.5% and 2.3% among patients with fungemia.^1^, ^2^ Limited data suggests the disease mostly spreads from an infectious source to its destination through central vasculature; peripheral cultures may be falsely negative.^3^ Previous reports of *Rhodotorula* infections have included sepsis, pneumonia, meningitis and endocarditis, typically in immunocompromised hosts.^3^, ^4^ To our knowledge, no case of Rhodotorula brain abscess in either an immunocompetent or immunocompromised host has yet been reported.^1^, ^5^, ^6^, ^7^ Here, we describe a case of a 40‐year‐old Caucasian woman with common variable immune deficiency (CVID) and brain abscesses due to both *Rhodotorula mucilaginosa* and *Nocardia abscessus*.

## 2 CASE HISTORY AND EXAMINATION

**FIGURE 1:**
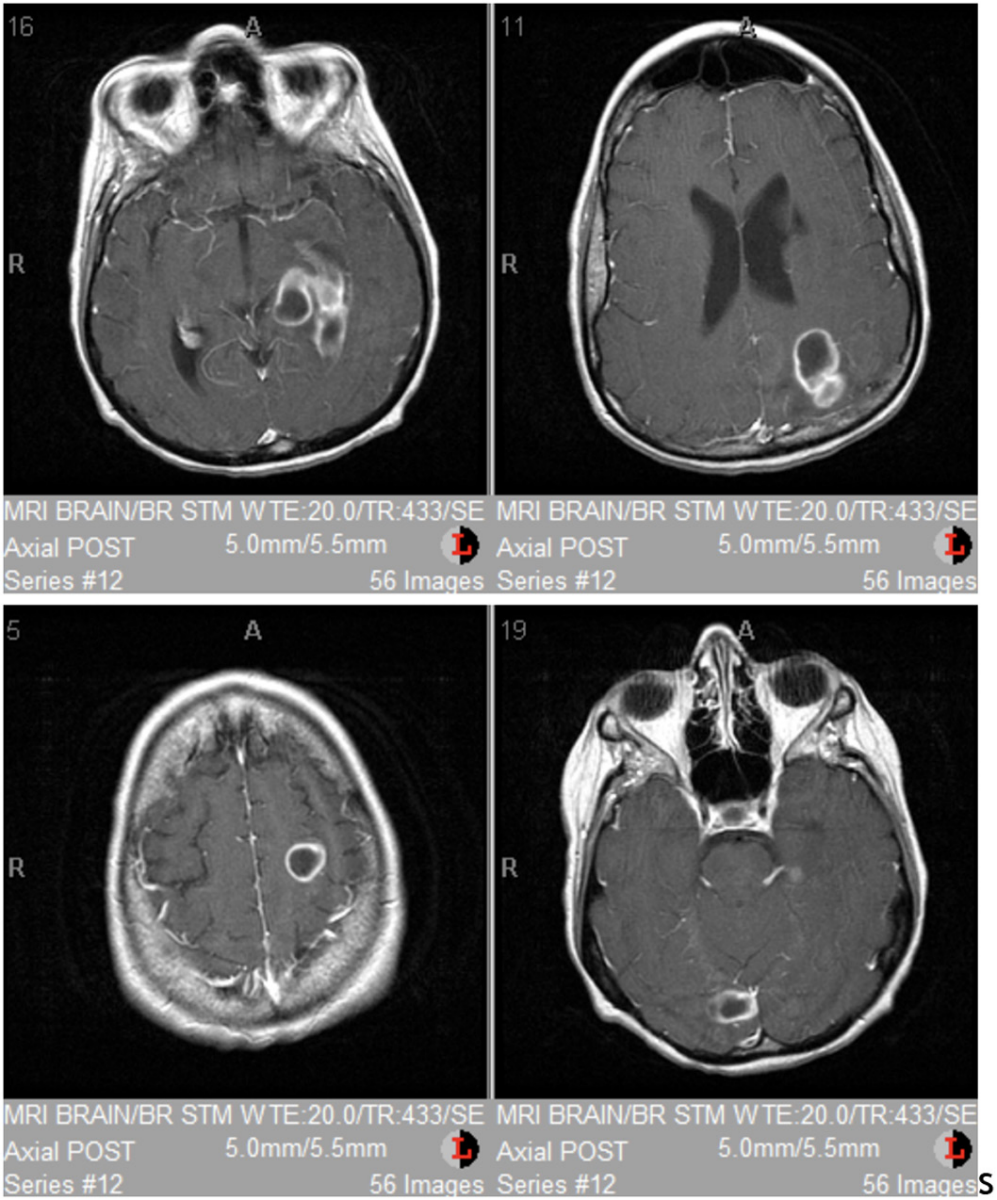
Brain MRI demonstrated intra‐axial ring‐enhancing lesions in the left posterior frontal lobe, left posterior parietal lobe, right occipital lobe and left temporal lobe, all with restricted diffusion and surrounding edema.

A 40‐year‐old Caucasian woman presented to the emergency department complaining of fevers, worsening shortness of breath, and new‐onset witnessed tonic–clonic seizures. Her medical history was significant for mental retardation, tobacco usage of 40 pack years, chronic obstructive pulmonary disease diagnosed in her 30s with a 2 L O2 home oxygen requirement, and multiple complicated previous hospitalizations for various infections. She also endorsed 2 months of unwitnessed syncopal episodes, headaches with intense pain over the left eye, worsening dyspnea with exertion (able to walk 15 feet from a baseline of 100), cough producing yellow sputum, and weight loss. She had no associated photophobia, neck rigidity, or blurred vision. On physical exam, she was febrile to 103°F, tachycardic, and hypoxemic despite home oxygen support. She had diffuse crackles throughout both lung fields; subsequent chest x‐ray showed expanded lung fields consistent with the patient's known COPD, and a diffuse reticulonodular infiltrate suggested by CT scan to be necrotizing pneumonia with cavitation. Brain MRI revealed multiple scattered intra‐axial ring‐enhancing lesions in the left posterior frontal lobe, left posterior parietal lobe, right occipital lobe and left temporal lobe, all with restricted diffusion, and surrounding edema (Figure 1).

## 3 DIFFERENTIAL DIAGNOSIS, INVESTIGATIONS, AND TREATMENT

**FIGURE 2:**
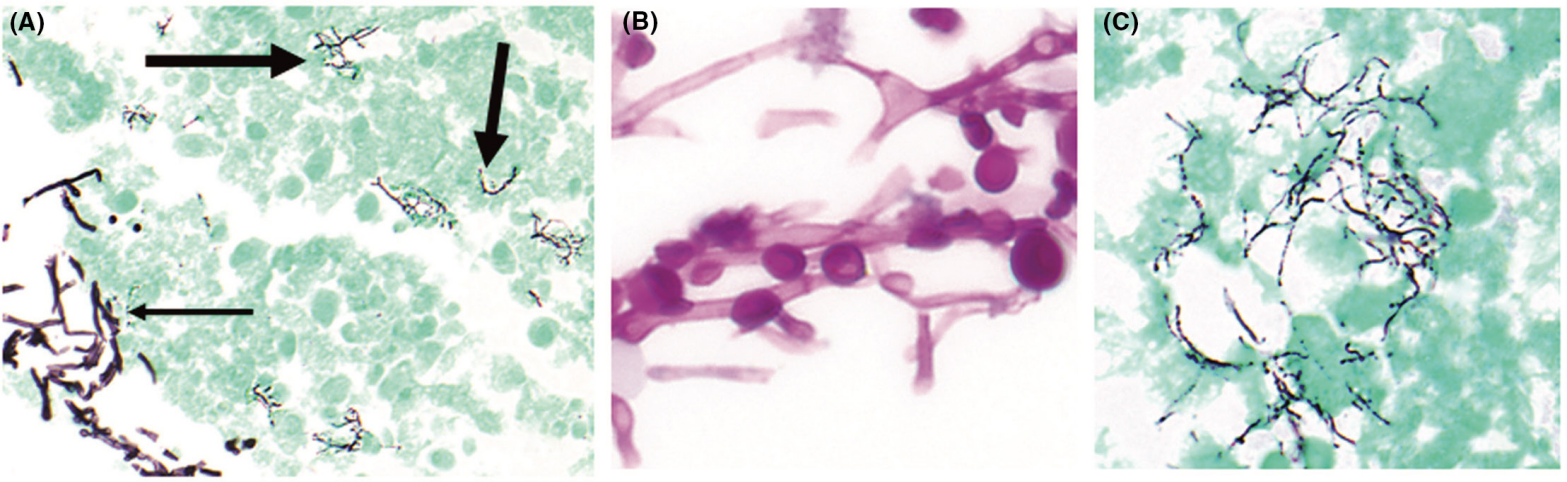
Histologic evaluation of brain tissue identified: (A) abundant necrotic debris and a chronic inflammatory cell infiltrate admixed with fungal forms (thin arrow) and filamentous bacteria (thick arrows) within the parenchyma (Gomori's methenamine silver (GMS) stain; 400 x magnification); (B) On higher magnification, hyphal forms with globose yeast cells (Periodic acid‐Schiff stain; 1000 x oil magnification) and (C) beaded, branching filamentous bacteria were seen (GMS; 1000 x oil magnification).

Given these findings, empiric antibiotic treatment was started for suspected brain abscess, but as metastatic disease could not be ruled out, brain, and transbronchial biopsies were performed. Histopathologic examination revealed a brain abscess with both budding yeast and filamentous bacilli (Figure 2), and cultures of the brain biopsy grew *R. mucilaginosa* and *N. abscessus*. Lung biopsy was unremarkable. Due to the rare, invasive fungal infection in the absence of known risk factors such as CVC, workup for immunocompromising conditions was undertaken, aspects of which are presented in Table 1. This workup eventually revealed a diagnosis of CVID. Empiric antibiotic therapy was thus replaced with meropenem, trimethoprim‐sulfamethoxazole, and liposomal amphotericin B. Amphotericin B associated electrolyte abnormalities prompted a switch to voriconazole, but unfortunately, despite several weeks of intensive medical therapy, the patient's respiratory status continued to decline, and she was transferred to the ICU for mechanical ventilation.

**TABLE 1:** The diagnostic workup leading to a diagnosis of CVID included multiple HIV tests, which were consistently negative, titers that remained low despite vaccinations, a positive mitogen induced lymphocyte blastogenesis test, and negative purine nucleoside phosphorylase and adenosine deaminase tests. Based on the patient's hypogammaglobulinemia, impaired antibody response, negative HIV test, and mitogen tests, the patient was believed to have common variable immune deficiency.

Lab		Result
HIV	PCR p24 viral load CD4	Negative Negative 0 47–198
Immunodeficiency	CD3 CD8	88 88
Immunization titers	Pneumococcal Diphtheria Tetanus	Undetectable 0.01 0.16
Immunoglobulins	IgG IgA IgM IgE	155 (nl > 400) 20 36 < 2
Mitogen induced lymphocyte blastogenesis Purine nucleoside phosphorylase assay Adenosine deaminase test		Markedly reduced Negative Negative

## 4 OUTCOME AND FOLLOW‐UP

After 2 weeks, the patient was still dependent upon mechanical ventilation and prognosis was felt to be poor. Tracheostomy was discussed with the patient's power of attorney, but after continued deterioration, comfort measures were prioritized, and the patient passed.

## 5 DISCUSSION

*Rhodotorula* remains a rare cause of infection. Previous reports of disease caused by *Rhodotorula* have primarily been found in immunocompromised hosts— such as the patient presented here with CVID— with a majority of these involving CVC—associated infections.^8^, ^9^, ^10^, ^11^ As it is still not clear why some immunocompromised patients develop complications such as *Rhodotorula* meningitis or endocarditis whereas others simply have fungemia, patient history, and clinical presentation alone may not converge on *Rhodotorula*.^12^, ^13^ Thus, histopathologic evaluation remains essential to confirming diagnosis and, as in this case, determining treatment course. There remains no definitive treatment algorithm for *Rhodotorula*: suggested treatments include simple supportive care, fluconazole, various formulations and dosing regimens of amphotericin B with or without flucytosine, and flucytosine/itraconazole combinations.^14^, ^15^ As seen in our patient, these regimens often cause complications due to their toxicities. Additionally, this patient's comorbidities, especially her coexisting respiratory failure, were felt to have contributed to her poor outcome. We hope the contribution of this novel manifestation of *Rhodotorula* as causing brain abscesses nonetheless serves as another datapoint to hopefully improving the diagnosis and management of this rare disease.

## AUTHOR CONTRIBUTIONS

**Arjun Bhatt:** Investigation; project administration; writing – original draft; writing – review and editing. **Melinda S. Dunalp:** Conceptualization; investigation; methodology; writing – review and editing. **Han Pham:** Conceptualization; investigation; methodology; resources. **Kimmo J. Hatanpaa:** Conceptualization; data curation; investigation; methodology; supervision; writing – review and editing. **Philipp Boyer:** Project administration. **Rita M. Gander:** Investigation; methodology. **Anna G. Symmes:** Supervision; writing – review and editing.

## FUNDING INFORMATION

The author(s) received no financial support for the research, authorship, and/or publication of this article.

## CONFLICT OF INTEREST STATEMENT

All authors declare that they have no conflicts of interest.

## CONSENT

Written informed consent was obtained from the patient to publish this report in accordance with the journal's patient consent policy.

## Data Availability

The data that support the findings of this study are available from the corresponding author upon reasonable request.
